# Likelihood-based optimization enables accurate copy number estimation for paralogous genes using exome data

**DOI:** 10.1093/bioinformatics/btag221

**Published:** 2026-07-07

**Authors:** Sang Yoon Byun, Vikas Bansal

**Affiliations:** Computer Science and Engineering, University of California San Diego, La Jolla, CA 92093, United States; Computer Science and Engineering, University of California San Diego, La Jolla, CA 92093, United States; School of Medicine, University of California San Diego, La Jolla, CA 92093, United States

## Abstract

**Motivation:**

Exome sequencing is widely used for genetic studies; however, accurate detection of copy number variants (CNV) in paralogous genes is challenging due to short-read mapping ambiguity and extensive copy-number variation. The human genome contains several hundred paralogous genes, many of which are known to harbor disease-associated CNVs. Existing exome CNV callers are primarily designed for rare CNV detection in uniquely mappable regions and are not well-suited for paralogous genes.

**Methods:**

We describe a computational method (EdgeCopy) for copy number profiling of paralogous genes using whole-exome sequence data. EdgeCopy aggregates reads mapped to all copies of paralogous genes and relates observed read depth to copy number for multiple exome samples using an approximate composite likelihood function. The likelihood function is optimized using numerical optimization to obtain gene-level fractional copy number estimates that are discretized and refined using a Hidden Markov Model to obtain exon-level copy number estimates.

**Results:**

Benchmarking of Edgecopy using experimental copy number data showed high concordance (mean* = *0.973) for six disease-associated paralogous genes. We evaluated performance using whole-exome data from approximately 2400 samples across five continental populations from the 1000 Genomes Project. EdgeCopy shows robust concordance with whole-genome sequencing based estimates (0.974–0.982) across populations and 130 paralogous genes spanning a wide range of copy-number variation. In comparison, copy number analysis using a state-of-the-art exome CNV caller failed to estimate copy number for paralogous genes with very high mapping ambiguity and showed much lower concordance (0.565) for CNV events compared to EdgeCopy (0.908).

**Availability:**

EdgeCopy is freely available at https://github.com/vibansal-lab/edgecopy.

## 1 Introduction

Segmental duplications or low copy repeats—long DNA sequences with two or more copies that share high sequence similarity—cover approximately 7% of the human genome (Sharp et al. 2005, Vollger et al. 2022). Compared to unique regions of the genome, segmental duplications are highly susceptible to copy number changes mediated by non-allelic homologous recombination (Sudmant et al. 2010). Several hundred protein-coding genes overlap segmental duplications and copy number variation in several such genes is associated with the risk of various diseases (Hollox and Hoh 2014). Copy number changes in the *SMN1* gene, which lies in a 100 kb long duplication with 99.9% sequence identity with its homologous region (Lefebvre et al. 1995), are known to cause spinal muscular atrophy, a severe pediatric disease. Furthermore, *SMN1* and its homologous gene *SMN2* show common copy number variation across human populations (Chen et al. 2020, Lopez-Lopez et al. 2020). Another notable example of a disease-associated paralogous gene is the *C4A/C4B* gene at the HLA locus. Copy number for two paralogous genes *C4A* and *C4B* varies from 2 to 8 in human populations (Sekar et al. 2016) and increased copy number of *C4A* is strongly associated with an increased risk of schizophrenia. Mandelker et al. (2016) identified 85 genes with short-read mapping ambiguity that are well-established disease genes. Experimental methods have been developed to assay copy number for a number of disease-relevant paralogous genes such as *SMN1/2*, *C4A/C4B*, and *FCGR3A/FCGR3B*.

Whole-genome sequencing (WGS) using short-read sequencing technologies such as Illumina can provide information about CNVs since read depth is linearly proportional to copy number. However, copy number analysis of segmental duplications is challenging due to ambiguity in read mapping between multiple homologous sequences. This problem can be overcome by jointly analyzing read counts mapped to paralogous copies of a segmental duplication. Sudmant et al. (2010) used Illumina WGS to generate the first genome-wide map of copy number variation in genes that overlap segmental duplications. They also showed that it is feasible to estimate total copy number and paralog-specific copy number of duplicated gene families. Similarly, Handsaker *et al.* developed the Genome STRiP method to analyze copy number variation in multi-copy genes using low-coverage WGS data from the 1000 Genomes Project (Handsaker et al. 2015). Computational tools for estimating copy number variation using WGS data in paralogous genes have continued to advance in recent years (Chen et al. 2020, Prodanov and Bansal 2022). Some of these tools are designed for individual genes that are medically relevant (Chen et al. 2020) (e.g., *SMN1/2*) while others are able to analyze different loci in the human genome (Prodanov and Bansal 2022).

Whole-exome sequencing (WES)—the targeted sequencing of protein-coding regions—is a widely used assay for DNA sequencing in human genetic studies (Bamshad et al. 2011, Gilissen et al. 2012). In comparison to WGS, WES is less costly since it targets only 2–3% of the genome. The most recent release of the gnomAD database of genetic variation contains more than ten times as many exomes (>700 000) as whole-genomes (Chen et al. 2024). However, exome sequencing provides high coverage for only exonic regions and the per-base read depth varies significantly across exons. The high exon-to-exon variance in coverage in exome sequencing makes it challenging to directly relate read depth to copy number. A number of read-depth based methods for detecting CNVs using WES data have been developed (Sathirapongsasuti et al. 2011, Fromer et al. 2012, Krumm et al. 2012, Plagnol et al. 2012, Babadi et al. 2023) since 2010. These methods leverage the fact that although absolute read depth varies tremendously across exons, samples sequenced using the same exome sequencing protocol show reproducible exon-specific coverage profiles. Exome CNV calling tools have been quite successful in identifying disease-relevant CNVs in large-scale genetic studies, nevertheless, exome CNV calling accuracy is lower than WGS-based CNV detection and new methods continue to be developed to improve accuracy (Mandiracioglu et al. 2024).

Existing methods for exome CNV calling have been designed for analysis of exons that are uniquely mappable using short reads (Babadi et al. 2023). CNV calling in paralogous genes poses several challenges that are not addressed by existing CNV calling methods. First, a large fraction of reads in low mappability exons (abundant in paralogous genes) cannot be mapped unambiguously and such reads are typically filtered out by CNV calling methods. In fact, many callers filter out segmental duplications and low-mappability exons prior to analysis (Babadi et al. 2023). Second, a significant fraction of paralogous genes show common variation in copy number (e.g., combined copy number for the *SMN1* and *SMN2* genes varies from 1 to 6 in humans (Butchbach 2016)) and accurately estimating the copy number requires differentiating between high copy number values. In contrast, exome CNV calling tools are primarily focused on detecting rare CNVs. Furthermore, many tools only report deletion/duplication events (Plagnol et al. 2012) and do not estimate integer copy number values, which makes them ill-suited for CNV analysis of paralogous genes. To the best of our knowledge, only one exome CNV calling method (CoNIFER) has been used to analyze copy number of paralogous genes (Krumm et al. 2012). Forni et al. (2015) used a customized version of CoNIFER to estimate copy number for the beta-defensin locus using exome data from the 1000 Genomes Project. However, this customized approach required manual clustering and cannot be applied to other paralogous genes.

We present a new computational method (EdgeCopy) designed to address the challenge of accurate copy number profiling of paralogous genes using exome data. To overcome the challenge of mapping ambiguity for short reads, it aggregates reads mapped to paralogous sequences and estimates the combined, or aggregate, copy number (e.g., for the *SMN1* and *SMN2* genes, it estimates *SMN1*+*SMN2* copy number). This is a widely used approach for WGS-based profiling of paralogous genes (Sudmant et al. 2010, Handsaker et al. 2015, Prodanov and Bansal 2022). To model the relationship between exonic read depth and copy number, EdgeCopy extends the beta-binomial read depth model from the ExomeDepth CNV calling method (Plagnol et al. 2012) and constructs a composite likelihood function that relates read counts for all samples to copy number values. The main novelty of our approach is the use of numerical optimization to optimize the composite likelihood function, enabling accurate copy number estimation for paralogous genes with rare and common CNVs. We perform comprehensive benchmarking of our method using WES data for 2400 samples from the 1000 Genomes Project and use experimental and WGS-based copy number estimates for assessing accuracy.

## 2 Methods

**Figure 1:**
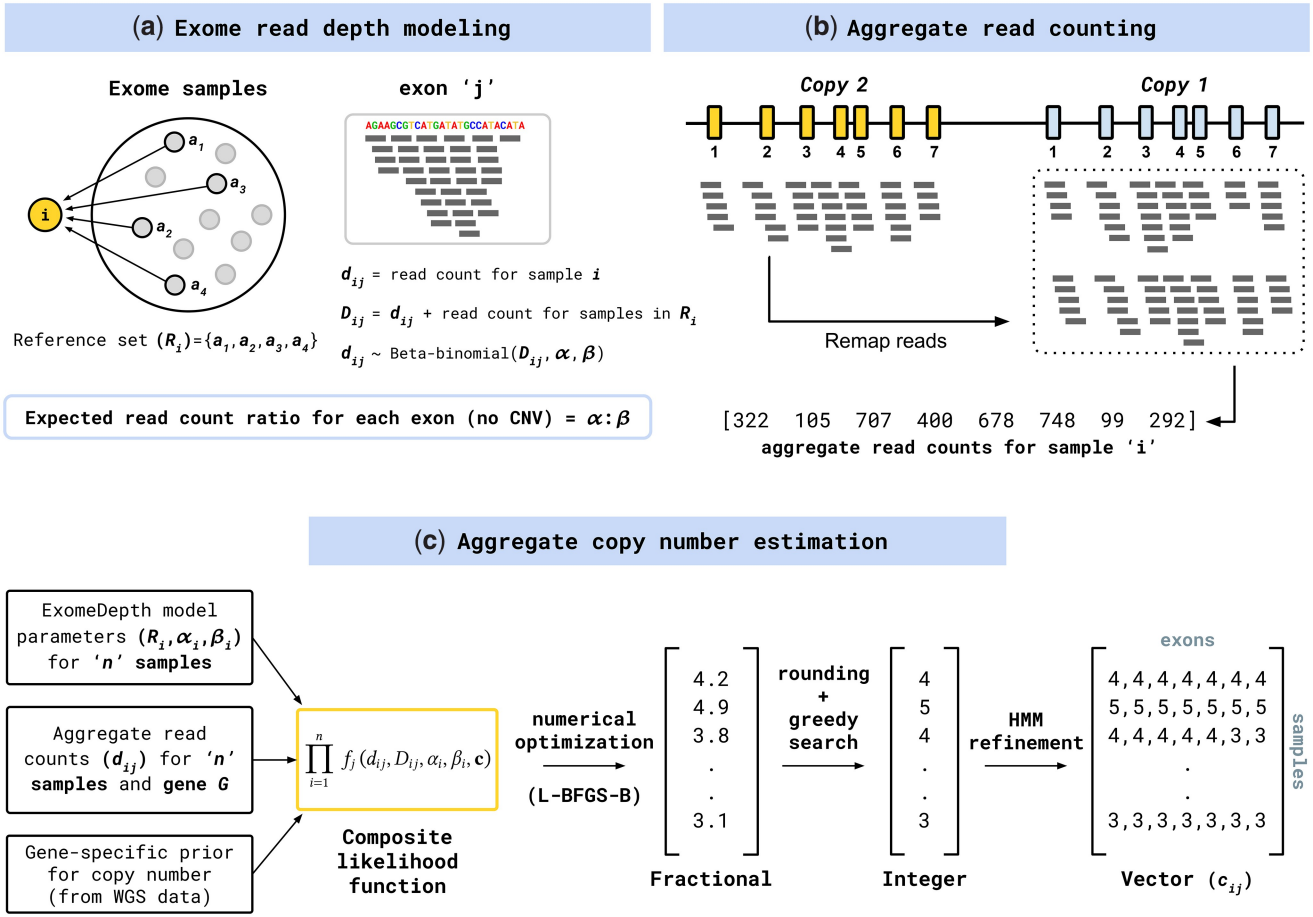
Overview of EdgeCopy method for read-depth based estimation of aggregate copy number for paralogous genes from exome data. (a) Read depth modeling using ExomeDepth: for each exome sample, a reference set $R_{i}$ is identified and a beta-binomial model is fit to model relative read counts for the sample and its reference set. (b) Aggregate read counting for paralogous genes: For a two-copy paralogous gene (with 7 exons), reads from the second copy are re-mapped to the first gene to calculate the aggregate per-exon read depth for each sample. (c) Joint estimation of aggregate copy number: aggregate read counts for each sample, prior copy number frequencies, and the ExomeDepth model parameters are used to construct the composite likelihood function. The composite likelihood function is optimized using a three-step approach: (i) numerical optimization, (ii) greedy local search and (iii) HMM refinement, to obtain per-exon copy number values for each sample.

Given exon-level read count data from *n* samples sequenced using exome capture, we consider the problem of estimating copy number for a gene with *m* exons. Specifically, let $d_{ij}$ denote the read count for sample *i* at exon *j* of a specific gene. Using the observed read count matrix, we seek to infer the copy number estimates $c_{ij}(1\leq i\leq n$ and 1≤j≤m) that best explain the observed read counts under a probabilistic model. The following sections introduce the exome read depth modeling framework, the likelihood formulation and the optimization methods used in our method EdgeCopy. Subsequently, we describe the copy number estimation algorithm obtained by integrating these components (see Fig. 1 for an overview).

### 2.1 Read depth modeling

Unlike whole-genome sequencing, exome sequencing does not yield a stable per-exon coverage baseline from an individual sample; therefore, read depth for each sample is typically modeled using other samples (sequenced using the same protocol) as a reference. We use ExomeDepth (Plagnol et al. 2012)’s approach of identifying a “reference set” of exome samples for each exome sample and modeling read depth for a sample relative to other samples using a beta-binomial distribution.

Let *i* denote a test sample and $R_{i}$ the “reference set” of samples for that sample. For exon *j*, let $D_{ij}=d_{ij}+\sum_{r\in R_{i}}d_{rj}$ be the total read depth for the reference set samples and sample *i*. The key idea is that although $d_{ij}$ varies across exons, the expected value of the fraction of reads for sample *i* ($p_{ij}=d_{ij}/D_{ij}$) is constant across exons since it primarily depends on the exon-specific capture efficiency. ExomeDepth finds a reference set that maximizes power to detect CNV events (Plagnol et al. 2012) and fits a beta-binomial distribution to the observed read counts ($d_{ij}$ and $D_{ij}$) using a sufficiently large number of exons (default = 10 000). Using a beta-binomial distribution allows for modeling overdispersion in the read counts due to variation in GC content, exon length, etc. Let $\alpha_{i}$ and $\beta_{i}$ be the estimated parameters of the beta-binomial distribution for sample *i*. Then for any exon *j*,


$$\mathrm{Pr}(d_{ij}|D_{ij},\alpha_{i},\beta_{i})=\left(\begin{array}{c} D_{ij}\\ d_{ij} \end{array}\right)\frac{B(d_{ij}+\alpha_{i},\;D_{ij}-d_{ij}+\beta_{i})}{B(\alpha_{i},\beta_{i})}$$

Where B(·,·) is the beta function. This relates the read depth $d_{ij}$ to the corresponding read depth for its reference set ($R_{i}$) under the assumption that the copy number of all samples is 2. Intuitively, the $\alpha_{i}$ : $\beta_{i}$ ratio determines the expected sample-to-reference read count ratio for any exon. An increase (or decrease) in copy number for sample *i* (or one or more samples in the reference set) is likely to shift the observed read count ratio. To model copy number changes, we define a scaled beta-binomial distribution that relates $d_{ij}$ and $D_{ij}$ for arbitrary copy number values. Let $c=\{c_{ij}:i=1,\ldots ,n,\;j=1,\ldots m\}$. Let $m_{k}$ be the mean read depth for sample *k* (average across all exons). We define two parameters $s_{1}$ and $s_{2}$ as follows:


$$s_{1}=\alpha_{i}\cdot c_{ij},\;s_{2}=\beta_{i}\cdot \frac{\sum_{k\in R_{i}}c_{kj}m_{k}}{\sum_{k\in R_{i}}m_{k}}$$

To keep the overdispersion ($\frac{1}{\alpha_{i}+\beta_{i}}$) of the scaled beta-binomial distribution unchanged, we scale the parameters using the sum $\alpha_{i}+\beta_{i}$. Using $s_{1}$ and $s_{2}$, we can define the parameters of the scaled beta-binomial distribution that depend on the copy number of sample *i* and samples in $R_{i}$ as:


$$\alpha_{i}(c)=\frac{s_{1}}{s_{1}+s_{2}}(\alpha_{i}+\beta_{i}),\;\beta_{i}(c)=\frac{s_{2}}{s_{1}+s_{2}}(\alpha_{i}+\beta_{i})$$

Now we can define a likelihood of the observed read counts for sample *i* as a function of the copy number of sample *i* and samples in the reference set $R_{i}$:


$$f\left(d_{ij},D_{ij},\alpha_{i},\beta_{i},c\right)=\mathrm{Pr}(d_{ij}|D_{ij},\alpha_{i}(c),\beta_{i}(c))$$

ExomeDepth (Plagnol et al. 2012) assumes that the copy number of the reference set samples is equal to the reference copy number and uses a Hidden Markov Model (HMM) framework to detect copy number changes for each sample and each gene using the beta-binomial likelihood. While this approach works for genes with rare CNVs, it does not correctly model genes with common CNVs. Therefore, we define a single likelihood function that relates the read counts for all samples to the copy number values.

### 2.2 Composite likelihood function

The likelihood for sample *i* depends on the copy number for *i* and also samples in the reference set $R_{i}$. We define an approximate composite likelihood function for exon *j* as:


$$L_{j}\left(c\right)=\prod_{i=1}^{n}f\left(d_{ij},D_{ij},\alpha_{i},\beta_{i},c\right)$$

If we assume that copy number is constant across a gene for each sample, the composite likelihood function over all exons for a gene can be written as:


$$L\left(c\right)=\prod_{j=1}^{m}L_{j}\left(c\right)$$

### 2.3 Maximizing likelihood function

The likelihood term for each sample depends on copy-number state of *i* and samples in $R_{i}$ (typical size = 10–15 for ExomeDepth). The reference-set structure induces a graph over samples in which edges connect samples appearing in each other’s likelihood terms. Copy-number states for samples in connected components of this graph are jointly constrained and optimization of the likelihood function can be used to infer them. However, maximizing the composite likelihood function is computationally challenging—even if we assume that copy number for each sample is constant across a gene—due to the discrete nature of the copy number values. To facilitate optimization, we can relax the constraint that copy number values are integers and allow them to take fractional values. Under this relaxation, the corresponding log-likelihood is a smooth, differentiable function of c and can be potentially optimized using a bounded quasi-Newton optimization procedure such as L-BFGS-B (Byrd et al. 1995). However, the relaxed likelihood function depends on c only through relative copy numbers since $\alpha_{i}(c)$ (and $\beta_{i}(c)$) depend on the ratios of $s_{1}$ and $s_{2}$. Therefore, it is invariant to rescaling, i.e. L(c)=L(θc) for θ>0. To resolve the scale invariance of the copy number, we introduce an informative prior on copy-number values derived using empirical data from whole-genome sequencing (WGS).

Let p(c) denote the empirical frequencies of integer copy-number states *c* estimated from WGS data for a specific gene. We define a prior density (π(c)) over continuous copy-number values $c\in R_{+}$ as a weighted mixture of Gaussian distributions,


$$\pi (c)=\sum_{k=0}^{C_{\max}}p(k)N\left(c|k,\sigma^{2}\right),$$

Where $\sigma^{2}$ controls the degree of smoothing around integer copy-number states and $C_{\max}$ is an upper bound on the copy number for a specific gene. By concentrating mass near integer copy-number values, the prior encourages fractional solutions close to integers, enabling simple rounding to generate an initial integer-valued solution. For each gene, the copy-number prior was derived using WGS based copy number estimates from populations distinct from the target population (e.g., average of all non-European populations for European population). The standard deviation (σ) for the Gaussian distributions was set to 0.35, allowing modest overlap between adjacent copy number distributions while still favoring values close to integers. We used the SciPy implementation of L-BFGS-B (scipy.optimize.minimize), using default optimization parameters. Copy-number variables were constrained to the interval [0.05,10].

### 2.4 Greedy heuristic for gene-level copy number

Fractional copy-number estimates obtained from numerical optimization are converted to integer copy numbers by rounding to the nearest integer. This integer-valued copy-number vector is then used to initialize a local greedy search aimed at identifying higher-likelihood solutions. At each iteration of the greedy search, the copy-number state of a single sample is updated to the value that maximizes the likelihood function, while copy-number states of all other samples are held fixed. The procedure is repeated until the overall likelihood stops increasing.

Let $\hat{c}$ be the integer copy number vector from the greedy heuristic. To estimate a quality value for sample *i’*s copy number estimate, we approximate the marginal posterior using a restricted sum over copy number vectors in a local neighborhood $S_{i}$ of $\hat{c}$ that includes copy number vectors that differ only at position *i* and vectors such that the copy number of all samples in $i\cup R_{i}$ is increased or decreased by 1 relative to $\hat{c}$. Empirical analyses showed that restricting the marginalization to this reference-set–based neighborhood captures the dominant posterior mass.

### 2.5 Exon-level copy number refinement using an HMM

Next, to obtain exon-level copy-number estimates for each sample, we applied a Hidden Markov Model (HMM) to refine the integer copy-number configuration inferred at the gene level. The greedy heuristic yields a constant copy-number state across all exons of a gene for each sample; the HMM is used to explore nearby copy-number configurations and to detect partial copy number changes using this as the baseline. For each sample, the observed data consist of the aggregated read depth at each exon, and the hidden states correspond to the set of possible copy-number values at each exon. The specification of the initial, transition, and emission probabilities closely follows that of ExomeDepth (Plagnol et al. 2012), with minor modifications to the emission model to use the composite likelihood function (described in detail in the Supplementary Data, available as supplementary data at *Bioinformatics* online).

### 2.6 Aggregate read counts for paralogous genes

We define a paralogous gene *G* with *l* paralogous copies in the reference genome as a collection of *l* homologous loci (including functional genes and pseudogenes) that share high sequence similarity and for which a one-to-one correspondence can be established across a subset of exons. We define the aggregate copy number of an exon of a paralogous gene as the sum of the copy numbers of the homologous intervals across the *l* copies. To utilize all reads for estimating copy number, we obtain aggregate read counts by remapping the reads from all copies to a single representative copy (illustrated in Fig. 1B for a 2-copy gene). Homologous regions are identified using the Parascopy homology table (Prodanov and Bansal 2022). The read counts for each exon are calculated using the re-mapped reads (using all reads irrespective of mapping quality). For a paralogous gene, the *reference copy number* is defined as 2 times the number of repeat copies. Next, we describe the EdgeCopy algorithm for estimating aggregate copy number for a paralogous gene using aligned reads for *n* exome samples.

### 2.7 EdgeCopy algorithm

First, the ExomeDepth tool (Plagnol et al. 2012) is used to estimate the reference set ($R_{i}$) and beta-binomial parameters ($\alpha_{i}$ and $\beta_{i}$) for each sample *i* (see Fig. 1A for an illustration). The beta-binomial distribution is fit using read counts for uniquely mappable exons using default parameters. This step is performed only once before analyzing the copy number of paralogous genes.

Subsequently, for each paralogous gene, aggregate read counts for each sample are calculated by re-mapping reads from all repeat copies to a single copy (Fig. 1B). This generates the read count matrix $d_{ij}$ and aggregate copy number estimation is performed by optimizing the composite likelihood function in a series of three steps (illustrated in Fig. 1C):

1. Maximize the composite likelihood function using numerical optimization (L-BFGS-B) to obtain fractional CN estimates
2. Use a local greedy heuristic to infer the most likely copy number estimate (constant gene-level estimate for each sample) and quality values for each sample
3. Use a Hidden Markov Model to infer the most likely copy number vector for each sample (among vectors in the neighborhood of the copy number estimated from the greedy heuristic)

Once the aggregate copy number vector has been estimated for each sample, we can use the allele-specific read counts for paralogous sequence variants or PSVs to estimate paralog-specific copy number. Consider a bi-allelic PSV with two alleles *a* and *b* that differentiate between the different copies of a paralogous gene *G*. Let *c* be the aggregate copy number of the exon overlapping the PSV in a specific sample and the number of reads supporting the two alleles *a* and *b* be $n_{a}$ and $n_{b}$ respectively. Then the probability that the allele-specific copy number is (k,c−k), 0≤k≤c for the PSV can be calculated using the Binomial probability mass function (p=k/c):


$$p(n_{a},n_{b}|(k,c-k))=\left(\begin{array}{c} n_{a}+n_{b}\\ n_{a} \end{array}\right)p^{n_{a}}{\left(1-p\right)}^{n_{b}}$$

### 2.8 1000 Genomes exome data for benchmarking

To evaluate the method, we utilized whole-exome sequence (WES) data available from the 1000 Genomes Project (1000 Genomes Project Consortium *et al.* 2015). This dataset contains exome data for 2,691 individuals from five continental populations: AFR (African), AMR (American), EAS (East Asian), EUR (European) and SAS (South Asian). CRAM files (mapped to the GRCh38 reference genome) of exome samples from these five populations were downloaded from the 1000 Genomes Project ftp site. The exome data was generated using multiple exome capture kits (Agilent SureSelect and Nimblegen) across four different sequencing centers (BI, BGI, BCM and WUGSC). Each sub-group of samples (from same population and sequenced at the same center) was analyzed together in a single run of EdgeCopy. Copy number calls for 1000 Genomes samples obtained using Parascopy (Byrska-Bishop et al. 2022) on high-coverage WGS data were used for assessing accuracy of exome-based copy number estimates. 2,397 of the 2,691 samples had both exome and WGS data available and were used for concordance estimates.

### 2.9 Genes for benchmarking

To identify paralogous genes for benchmarking, we identified exons in the human genome (chromosomes 1–22) that share high sequence similarity with one or more genomic regions (using Parascopy’s homology table (Prodanov and Bansal 2022)). The exons were grouped into contiguous regions and clustered. This resulted in 334 gene clusters where for each gene, at least one exon had high sequence similarity (>98%). These included 119 two-copy gene pairs (e.g. *SMN1/SMN2*), 14 three-copy gene clusters, and 103 genes for which 80% or more of the exons were homologous with non-exonic regions. Next, genes were excluded if they: (i) consisted of a single exon, (ii) had less than two exons overlapping segmental duplications, (iii) contained exons overlapping more than two distinct segmental duplications, or (iv) were not covered in the exome capture kits used for sequencing in the 1000 Genomes Project (intersection with bed files). Genes with more than three copies were excluded since estimating the copy number of such genes requires differentiating between very high values (e.g., 7 and 8) which is challenging using exome data. We note that some genes (e.g., *OTOA*) which only partially overlapped a segmental duplication were also included. The final set of 130 paralogous genes was used for benchmarking.

We used experimental copy number data for six paralogous genes (*SMN1*, *C4A*, *FCGR3A*, *RHD*, Beta-defensin genes, *APOBEC3A*) from published studies that had used samples from the 1000 Genomes Project or the HapMap Project for genotyping. The copy number data was generated using different experimental techniques such as MLPA (Vijzelaar et al. 2019) or PRT (Hardwick et al. 2011). For each gene, we used samples overlapping between each study and the 1000 Genomes WES dataset for benchmarking.

### 2.10 Calculating concordance of exome copy number estimates

For the genes with experimental copy number datasets, accuracy was defined as the fraction of exons for which the exome-based copy number estimate was identical to the gene-level experimental value (averaged across samples). For assessing the accuracy of exome-based aggregate copy number estimates across the larger set of paralogous genes, we used high-coverage WGS data for the same set of samples from the 1000 Genomes Project (Byrska-Bishop et al. 2022). Copy number estimates for the WGS data previously generated using Parascopy (Prodanov and Bansal 2022) were used for calculating concordance. The concordance of WES copy number values for a given gene and sample was defined as the fraction of copy number estimates that matched the corresponding WGS values across the exons. For each gene, samples for which the WGS aggregate copy number was equal to the reference copy number (across all exons) were classified as ‘reference’ and the remaining samples as ‘non-reference’. For each gene, we computed the average concordance for all samples and the subset of samples with non-reference (deletion and duplication) WGS copy number values. For all copy number estimates generated by EdgeCopy, we used a default quality threshold (Phred scale) of 20. In other words, any exon-level copy number estimate with a quality less than 20 was considered as ‘NoCall’. Furthermore, if a sample’s integer copy number estimate for a given gene had a quality less than 20, its vector copy number estimate, across exons, was also categorized as ‘NoCall’. Additionally, genes with more than 20% of samples categorized as ‘NoCall’ were excluded from further analysis. A similar quality threshold of 20 was used for filtering WGS-based copy number estimates.

### 2.11 Comparison to existing exome CNV calling methods

We considered several existing exome CNV calling methods for benchmarking with EdgeCopy but found them unsuitable for copy number analysis of paralogous genes. Many tools either (i) do not report explicit integer copy number estimates (e.g., ExomeDepth, XHMM, ECOLE), or (ii) are primarily designed for detecting rare CNVs in uniquely mappable regions. Furthermore, many tools such as GATK-gCNV explicitly filter low-mappability exons. These design choices limit their applicability to paralogous genes, where ambiguous read mapping and common copy-number variation are widely prevalent (see Supplementary Data for more details, available as supplementary data at *Bioinformatics* online). Therefore, we utilized two custom approaches for comparison with EdgeCopy.

First, we re-implemented the ExomeDepth method (Plagnol et al. 2012), modifying it to use aggregate read counts for paralogous genes while inferring discrete copy number values (the original implementation only outputs deletion/duplication events). The beta-binomial model for estimating reference sets from the original method was used as is. A vector of reference copy numbers (4 or 6 for 2 and 3 copy genes) was used as the initial state for the HMM-based estimation.

Second, we used the GATK-gCNV (Babadi et al. 2023) exome CNV calling method to obtain aggregate copy number estimates for paralogous genes by independently analyzing each paralog (using default parameters) and subsequently combining the copy number estimates for paralogous exons. For this, we disabled GATK-gCNV pipeline filters that exclude low-mappability regions (details provided in Supplementary Data, available as supplementary data at *Bioinformatics* online).

## 3 Results

### 3.1 Accuracy on simulated data

**Figure 2:**
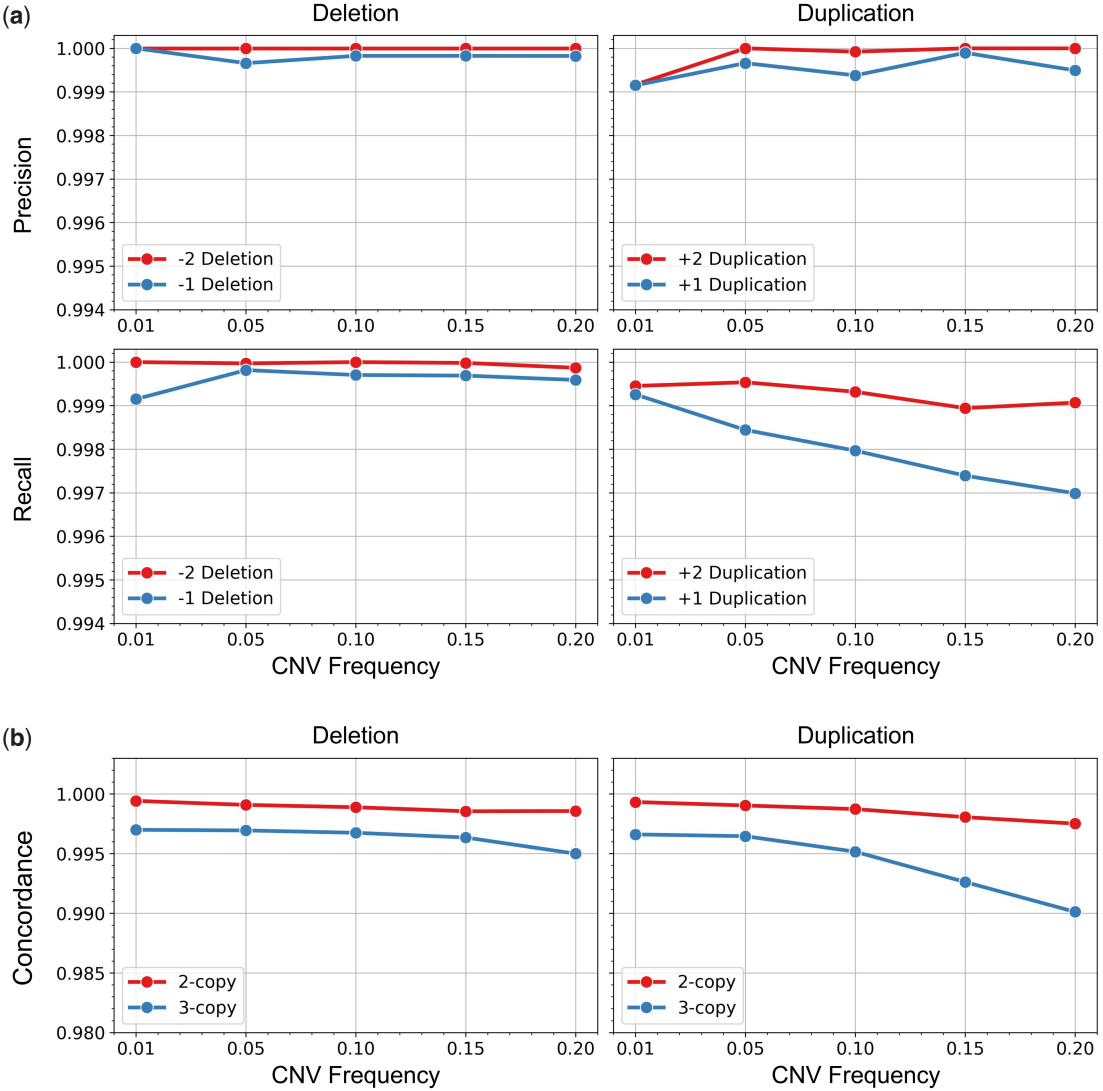
Accuracy of EdgeCopy on simulated CNVs. (a) Precision and recall for simulated deletions and duplications (copy number changes of −2, −1, +1, and +2 relative to the reference copy number) using EdgeCopy. For each simulation experiment, the fraction of samples with the CN change was varied from 0.01 to 0.20. (b) Comparison of accuracy for copy number genotyping for 2-copy and 3-copy paralogous genes (+1 and −1 copy number changes only).

The performance of EdgeCopy depends on a number of factors, including but not limited to the number of paralogous gene copies, number of exons, CNV population frequency, and sequencing depth in exome sequencing. We used simulations using read count data to assess how these parameters impact accuracy. For simulations, we utilized exome read count data for two (*n = *24) and three (*n = *3) copy paralogous genes from EUR ancestry samples that had either no CNVs or less than 3 samples with CNVs. Using the “null” read count matrix, we spiked in gene-level CNVs of varying frequencies and types (deletion and duplications). To simulate heterozygous deletions (or duplications) in a given sample, we used a binomial distribution to decrease (or increase) the raw depth of coverage for all exons of the gene with p=1/4 for 2-copy genes and p=1/6 for 3-copy genes. We evaluated five different CNV frequencies (0.01, 0.05, 0.10, 0.15 and 0.20) and four different CNV events (+1 duplication, +2 duplication, −1 deletion, −2 deletion). For each configuration, 10 independent trials were performed. Simulation results showed that EdgeCopy had high precision and recall (>99%) for detecting both deletions and duplications in 2-copy paralogous genes across a range of population frequencies (Fig. 2a). Notably, the accuracy for detecting duplications was marginally lower than that for deletions. The copy number estimation accuracy (measured as concordance) for 3-copy genes was high but slightly lower than that for 2-copy genes (Fig. 2b).

### 3.2 Benchmarking using experimental data

**Figure 3:**
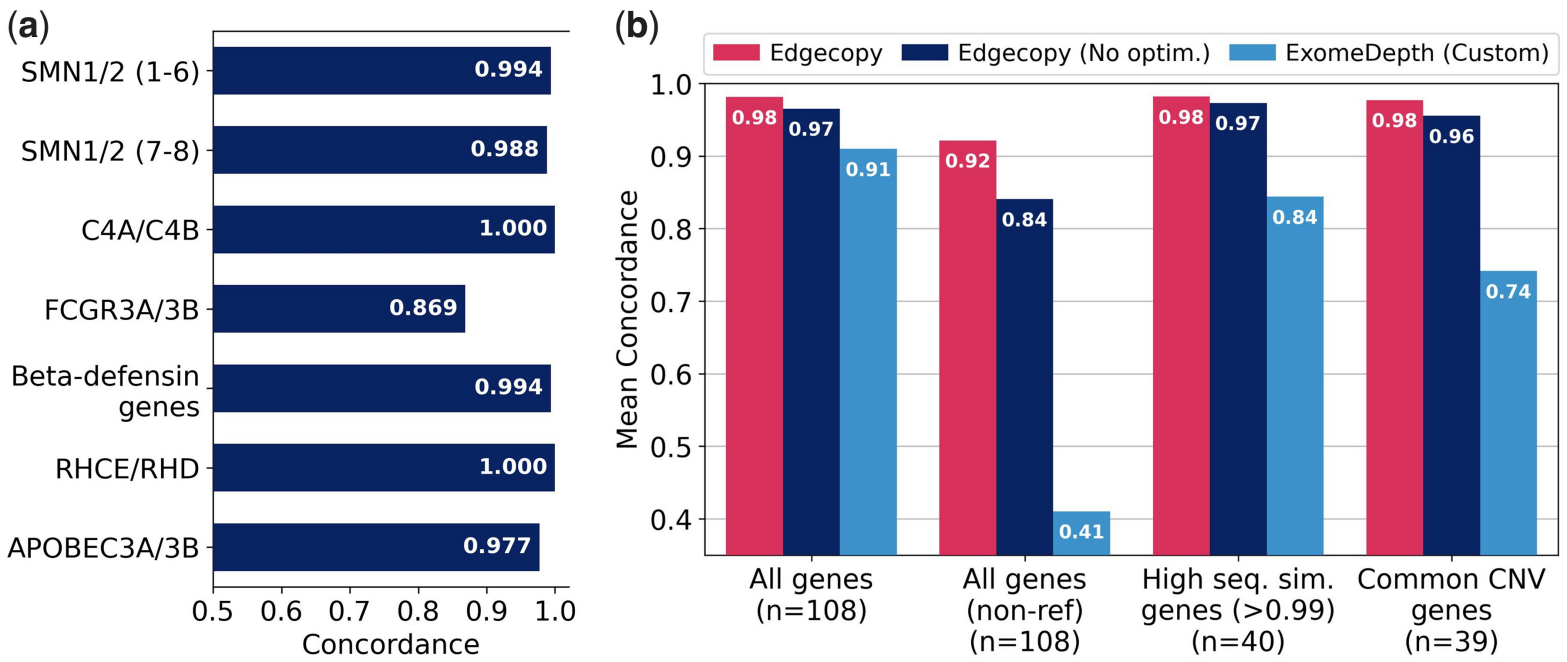
Accuracy of exome copy number estimates across methods and validation datasets. (a) Concordance between exome copy number estimates using EdgeCopy and experimentally derived copy number measurements for six paralogous genes. (b) Concordance between exome-derived copy number estimates and WGS–based estimates is shown for EdgeCopy, EdgeCopy without fractional optimization, and a custom ExomeDepth implementation across different gene subsets for EUR ancestry exome samples. ‘Non-reference’ refers to concordance for the subset of samples with non-reference WGS copy number.

First, we benchmarked the accuracy of each method on six paralogous genes with experimentally determined copy number data (Fig. 3a). The mean concordance between EdgeCopy and experimental estimates across the six genes was 0.973. In comparison, the mean concordance for the custom ExomeDepth implementation was 0.779 (Supplementary Table 1, available as supplementary data at *Bioinformatics* online). Among these genes, *SMN1/2* had the largest number of samples (1,121) with copy number values from the MLPA assay (Vijzelaar et al. 2019). For *SMN1/2*, EdgeCopy had a genotyping accuracy of 0.994 for exons 1–6 and 0.988 for exons 7–8 (Fig. 3a). Exons 7–8 harbor a common deletion event in European populations, and 54 of the 55 discordant samples had a deletion event that was not detected by EdgeCopy. The copy number concordance for the *C4A* (*n = *45) and *RHD* (*n = *36) paralogous genes was 100%. The *FCGR3A/3B* gene had the lowest accuracy for EdgeCopy with 16/122 discordant samples. For all 16 discordant samples, the EdgeCopy copy number estimates matched the WGS estimates suggesting that the experimental data may not be accurate. Notably, the experimental copy number values for this gene were obtained by combining three different assays since the individual assays had low accuracy (Hollox et al. 2009).

Finally, for the beta-defensin genes on chromosome 8p23.1 (six paralogous genes), in addition to the experimental copy number values (generated using the Nanostring nCounter system), Forni et al. (2015) built a custom pipeline using the output from the CoNIFER exome CNV calling tool to estimate aggregate copy number values from 1285 exome samples in the 1000 Genomes Project. Among 112 samples with CN calls from all methods, 4/112 calls from EdgeCopy were discordant with the Nanostring experimental estimates. In comparison, 6/112 calls from the custom CoNIFER-based pipeline were discordant. This demonstrated that EdgeCopy achieved higher accuracy than a custom exome copy number genotyping method.

### 3.3 Benchmarking accuracy across 130 paralogous genes

Next, we evaluated the performance of our method using a larger set of paralogous genes. We focused on genes covered by probes in exome sequencing and genes with high sequence similarity (>99%) that are likely to be challenging for existing methods. We compiled a list of 130 paralogous genes (see Methods) and used 483 European (EUR) ancestry samples from the 1000 Genomes Project for benchmarking using concordance with WGS-based estimates. 21 genes were filtered out due to a high rate (>20%) of samples with low-confidence copy number estimates while one gene did not have WGS copy number estimates. For the remaining 108 genes, the mean concordance between EdgeCopy’s estimates and WGS-based estimates was 0.981 (Fig. 3b). For samples with non-reference copy number (deletions and duplications), the average concordance was 0.921. The concordance of EdgeCopy for genes with high sequence similarity (>0.99 sequence similarity) and genes with common CNVs (genes with 5% or more samples having non-reference copy number in WGS data) was almost unchanged (Fig. 3b). In comparison, the custom implementation of the ExomeDepth method had much lower concordance for all samples (0.91) and particularly for samples with non-reference copy number values (0.41). We assessed whether per-sample concordance was dependent on sequencing depth and found no significant correlation with per-sample read depth (Spearman *r* = −0.065, *p* = 0.155, Supplementary Figure 1, available as supplementary data at *Bioinformatics* online).

EdgeCopy aggregates read depth from paralogous genes (exon-by-exon) to estimate aggregate copy number values. For genes with very high sequence similarity such as *SMN1/2* where 98.6% of reads had a mapping quality of zero in EUR exome data, diploid copy number calling that relies on reads mapped to each gene individually is unlikely to work. However, diploid copy number estimation could potentially work for genes with moderate mapping ambiguity. To assess the accuracy of diploid copy number estimation for paralogous genes, we estimated copy number for paralogous genes using the state-of-the-art tool GATK-gCNV (Babadi et al. 2023) (see Methods) by considering each gene copy as an independent gene and subsequently aggregated the copy number values for the paralogous exons. For this, we analyzed a subset of 79 paralogous genes where all copies correspond to protein-coding genes and all exons overlap segmental duplications. For 23 of the 79 genes (including genes such as *SMN1/2* and *C4A/C4B*), the GATK-gCNV pipeline did not report copy number estimates likely due to the high percentage of reads with low mapping quality or low read depth. For the remaining genes, the mean concordance with WGS estimates was 0.934 and 0.565 for all samples and samples with non-reference copy number (deletions and duplications) respectively. In comparison, EdgeCopy’s mean concordance values for the same subset of genes were 0.978 and 0.908. Even for the *RHD* gene (68% of reads with mapping quality of 20 or greater) GATK-gCNV estimates had low accuracy (concordance = 0.404). These results show that EdgeCopy’s approach to estimate aggregate copy number for paralogous genes achieves much higher accuracy.

### 3.4 Fractional copy number estimates and accuracy

**Figure 4:**
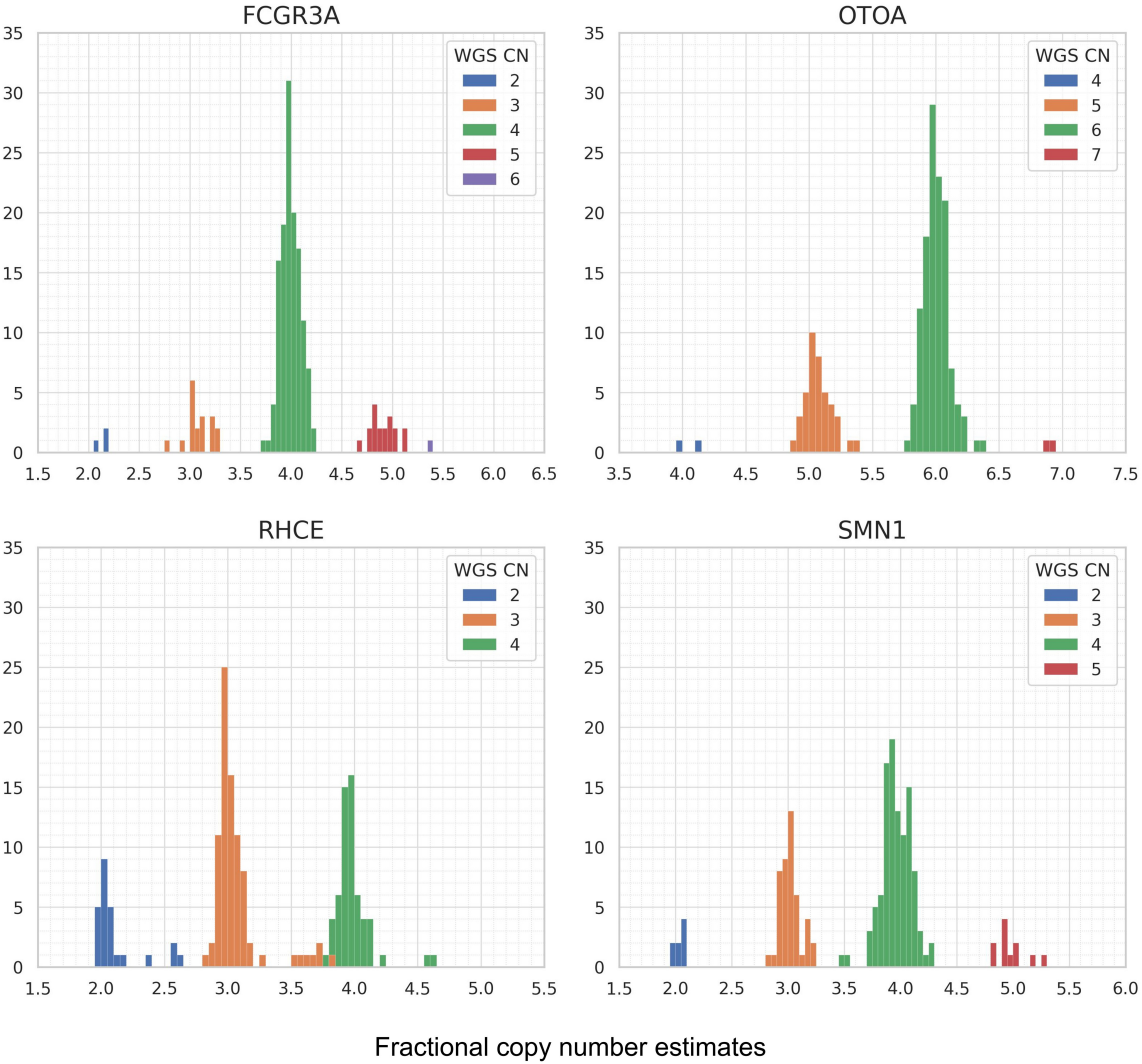
Distribution of fractional copy number estimates across four paralogous genes. Stacked histograms for fractional copy number values estimated using numerical optimization in EdgeCopy across 172 EUR samples are shown for four genes. The bars are color-coded by WGS-derived copy number, illustrating the correspondence between fractional estimates and integer copy number (CN) values from WGS data. The y-axis for each gene is the number of samples with the corresponding fractional copy number. The *OTOA* gene is a 3-copy paralogous gene while the other genes are 2-copy paralogous genes.

EdgeCopy uses numerical optimization of the composite likelihood function to first obtain fractional values of copy number estimates. Empirical results showed that the numerical optimization using L-BFGS-B was robust to initial copy number values, with convergence to identical or near-identical fractional solutions across diverse initializations (data not shown). We visualized the fractional copy number distributions for a subset of 172 EUR samples (sequenced at the BGI center using Agilent Sureselect exome kit) for four paralogous genes with common copy number variation (Fig. 4). The fractional copy number estimates form well-separated clusters that largely align with the WGS-based integer copy number values indicating that the numerical optimization captures the underlying discrete copy number values. To formally assess the importance of fractional copy number estimates for the overall accuracy of EdgeCopy, we performed an ablation study where fractional estimates were not used to generate the initial copy number vector. Rather, copy numbers of all samples were initialized to the reference copy number (4 or 6 for 2-copy and 3-copy paralogous genes) for the local greedy search heuristic. Across the 130 paralogous genes, removing the fractional optimization reduced the average concordance from 0.98 to 0.97 and the concordance for CNV events (non-reference copy number samples) from 0.92 to 0.84 (Fig. 3b).

### 3.5 Copy number accuracy across populations

Next, we analyzed WES data from four additional populations in the 1000 Genomes Project using EdgeCopy and estimated copy numbers for the same set of paralogous genes used for analyzing the EUR ancestry samples. For each population, we used WGS based estimates to calculate concordance for the EdgeCopy exome-based estimates. Across five continental populations, EdgeCopy estimates showed consistently high concordance with WGS estimates across 130 paralogous genes (Table 1). The average concordance across all genes was uniformly high (0.97–0.98), while concordance for samples with non-reference copy number values was lower but stable across populations (0.89–0.92). 16–23 genes per population were classified as ‘NoCall’ due to low-confidence estimates in more than 20% of samples, reflecting loci with ambiguous copy number inference. Further analysis revealed that 14 genes were consistently ‘NoCall’ across all populations due to either having a small number of exons, extensive variation in copy number or low coverage in specific exons. Among callable genes, the majority achieved very high gene-level concordance, with more than 80 (95) genes per population exhibiting a concordance of 0.99 (0.95) or more across all populations (Table 1).

**Table 1:** Concordance of exome copy number estimates across 130 paralogous genes and five continental populations.

Population	Samples	NoCall	Concordance	per-gene concordance (no. of genes)				
		genes		0.99–1	0.95–0.99	0.9–0.95	0.8–0.9	< 0.8
AFR	639	16	0.974 (0.895)	84	10	3	9	5
AMR	325	23	0.981 (0.905)	82	15	5	2	3
EAS	474	20	0.971 (0.884)	85	12	4	3	5
EUR	483	20	0.981 (0.921)	81	18	3	2	4
SAS	476	17	0.982 (0.915)	99	5	1	3	4

‘NoCall’ refers to genes with >20% samples with low confidence copy number values. ‘Concordance’ is calculated across all genes (the number in brackets is the concordance for samples with non-reference WGS copy number values). The number of genes with specific concordance values (five different bins) is shown for each population. The total number of genes is less than 130 for some populations since some genes did not have WGS based estimates available for comparison.

### 3.6 Paralog-specific copy number accuracy

To assess the accuracy of paralog-specific copy number estimation, we utilized experimental data for three paralogous genes (*SMN1*/*SMN2*, *C4A*/*C4B*, *FCGR3A*/*FCGR3B*) for which custom assays have been developed using specific PSVs (Hollox et al. 2009, Vijzelaar et al. 2019). We used these PSVs to estimate paralog-specific copy number from allele-specific read counts in exome data. The *SMN1* and *SMN2* genes differ by a single PSV (c.840C>T) in the coding region in exon 7. Among the 1,121 samples with MLPA based copy number estimates for *SMN1* (Vijzelaar et al. 2019), 1048 samples had matching aggregate copy number estimates. The concordance of exome based *SMN1*-specific copy number for these samples was 99.6%. For the *FCGR3A/3B* gene, among 98 samples for which the aggregate copy number matched experimental data, the allele-specific copy number values were concordant for 97 samples. For the *C4A/C4B* gene, the paralog-specific copy number concordance was 95.8% (46/48).

### 3.7 Run time and memory usage

For 483 exome samples from the European population, estimating the reference set and the beta-binomial parameters (using ExomeDepth) took 450 minutes using 16 threads on a Linux workstation (Ubuntu 18.04, 384 GB RAM, 24-core Intel Xeon 5220R CPU). Calculating the aggregate read counts and estimating the aggregate copy number for 130 genes using EdgeCopy took 350 minutes using 16 threads. The memory usage was less than 4 GB for all analyses. In comparison, GATK-gCNV took close to 405 hours of running time for analyzing the same set of European samples. The longer run time was partly due to the fact that GATK-gCNV builds the read depth model and calls CNVs simultaneously for all genes.

## 4 Discussion

Despite the availability of millions of human samples with exome sequence data, a subset of protein-coding genes that overlap segmental duplications remain largely unexamined for copy number variation. As a result, disease-associated CNVs in these genes, which show extensive copy number variation, remain under-explored. In this paper, we formulated exome-based copy-number estimation in paralogous genes as a likelihood optimization problem over exon-level read counts across samples. Our method addresses the two key challenges of analyzing paralogous genes using exome data: read mapping ambiguity and common copy number variation. It uses the core read-depth modeling approach from a previous exome CNV calling method (ExomeDepth) and a read aggregation approach that has been used in WGS studies for copy number analysis of paralogous genes (Prodanov and Bansal 2022).

Using numerical optimization to estimate fractional copy number values that provide the starting point for estimating integer copy number values is a key innovation of our method that enables accurate copy number estimation. To our knowledge, it is the first exome copy-number estimation approach to explicitly formulate copy-number estimation as a principled composite likelihood across samples. This approach could also enable accurate estimation for non-duplicated genes with common copy number variation (Babadi et al. 2023). EdgeCopy uses gene-specific priors obtained from WGS data to enable accurate copy number genotyping for paralogous genes that frequently show common copy number variation. For 1000 Genomes samples, population-independent copy number priors (derived from non-overlapping continental populations) were used for estimation, minimizing bias and demonstrating robustness of the method.

By comparison to WGS-based estimates, we show that EdgeCopy achieves greater than 99% concordance for the vast majority of paralogous genes. We find that a subset of paralogous genes cannot be genotyped with high confidence due to low depth of coverage, high variation in copy number and lower number of exons. Overall, accurate copy number estimation is achievable for most disease-relevant paralogous genes across diverse populations. Analysis of paralogous genes using a state-of-the-art exome CNV calling tool showed that many genes with very high mapping ambiguity cannot be genotyped, and that aggregating copy-number estimates across paralogous gene copies yields a concordance of 0.565, markedly lower than the concordance of 0.908 obtained using the read-aggregation approach of EdgeCopy.

The method presented in this paper complements existing exome CNV callers by enabling accurate copy number genotyping of paralogous genes. This will enable population genetic analyses and downstream association studies for paralogous genes using large-scale exome datasets available in databases such as the NIH dbGaP.

## Supplementary Material

btag221_Supplementary_Data

## Data Availability

All scripts, data files and WGS CNV calls used to generate the results in the paper are available at https://doi.org/10.5281/zenodo.19660291. Aligned whole-exome sequence data for samples from the 1000 Genomes Project is available from the 1000 Genomes project ftp site: https://doi.org/10.5281/zenodo.19660291https://ftp-trace.ncbi.nih.gov/1000genomes/ftp/

## Supplementary information

Supplementary figures and tables are available at *Bioinformatics* online.
