# Supplementary material for: Likelihood-based optimization enables accurate copy number estimation for paralogous genes using exome data
Source: Bioinformatics. 2026 Jul 7;42(Suppl 1):btag221. doi: 10.1093/bioinformatics/btag221 (PMC13340216; doi:10.1093/bioinformatics/btag221)
Supplement: btag221_Supplementary_Data [file btag221_supplementary_data.pdf]

Supplementary Materials for "Likelihood-based optimization enables accurate copy number estimation for paralogous genes using exome data"

Sang Yoon Byun and Vikas Bansal
ISMB 2026 Proceedings

Methods

HMM details

To model variation in copy number along the exons of a paralogous gene, we use a hidden Markov model (HMM) defined over exon indices (Plagnol *et al.*, 2012; Fromer *et al.*, 2012). For each sample, the hidden state $z_j \in \mathcal{C}$ represents the copy-number state for exon j, where $\mathcal{C}$ denotes the set of allowable copy numbers.

Initial probabilities:
The initial state distribution of the HMM was specified using empirical copy-number frequencies estimated from WGS data. Specifically, for each copy-number state $c \in \mathcal{C}$, the initial probability π_c was set proportional to its empirical frequency. If the specific copy number was not observed in WGS data, we use $\pi_c = 10^{-4}$.
Transition probabilities:
Let t denote the frequency of a CNV event (duplication or deletion). We disallow transitions between states that differ in copy number by more than two units. For current state c_t and new state c_s, where $|c_s - c_t| \leq 2$, we have the following transition probabilities:

$$p(c_t \to c_s) = \begin{cases} 1-t & \text{if } c_s = c_t \\ \frac{t}{4} & \text{if } c_s \neq c_t \end{cases}$$

The transition probabilities are normalized at gene boundaries to sum to 1. Similar to the ExomeDepth method (Plagnol *et al.*, 2012), we use $t = 10^{-4}$.
Emission probabilities:
Changing the copy number c_{ij} of exon j for sample i affects the likelihood for all samples that contain sample i in their reference sets. Therefore, the emission probability for sample i and exon j was earlier defined as follows:

$$f(d_{ij}, D_{ij}, \alpha_i, \beta_i, \mathbf{c}) \cdot \prod_{k \in S_i} f(d_{kj}, D_{kj}, \alpha_k, \beta_k, \mathbf{c})$$

where S_i is the set of samples that contain i in their reference set.
We use the Viterbi algorithm to estimate the most likely copy number vector for each sample in turn while keeping the copy number vectors for other samples fixed. This is repeated iteratively for all samples until the overall likelihood stops changing. Finally, the forward-backward algorithm is used to calculate the posterior probability distribution for the possible copy number values for each sample i and exon j.

Exome CNV calling methods and paralogous genes

A number of exome CNV calling methods infer copy number from read depth relative to a reference, but they differ substantially in how reference samples are selected, how overdispersion is handled, and how copy number states are inferred. Most existing approaches were developed with the implicit assumption that copy number variation is rare and that reads can be uniquely mapped to genomic loci, assumptions that are frequently violated in paralogous genes. Indeed, no previously published exome CNV calling method has explored the possibility of CNV calling or genotyping in paralogous genes except the CoNIFER method (Krumm *et al.*, 2012). Methods such as XHMM, Conifer, ExomeDepth and ECOLE report duplication/deletion output rather than integer CN estimates making them unsuitable for copy number estimation in paralogous genes where copy number values show wide variation.
Deep-learning–based approaches such as ECOLE frame exome CNV detection as a supervised classification problem, learning complex patterns in read depth across exons. These methods show improved performance in benchmark datasets with labeled CNVs. However, their applicability to paralogous genes has not been evaluated.

Estimating copy number for paralogous genes using GATK-gCNV
The recently published GATK-gCNV caller (Babadi *et al.*, 2023) use probabilistic frameworks to infer integer CN states, incorporating overdispersion and cohort-level effects. We used GATK-gCNV to assess if aggregate copy number for paralogous genes can be estimated by analyzing each copy of the paralogous gene independently (as a distinct non-duplicated gene). Only paralogous genes where each of the paralog copies was a coding gene were analyzed. Exonic intervals for genes located on chromosomes 1-22 were provided in Picard-style interval list format. When using the default annotation-based filters (e.g., segmental duplication and low-mappability tracks) in combination with the default count-based filters, GATK-gCNV excluded 104 of 130 (80.0%) paralogous genes and 91.8% of duplicated exons. To enable analysis of genes located in low-mappability regions, segmental duplication and low-mappability tracks were intentionally excluded from the AnnotateIntervals step. Even after disabling annotation-based filters, GATK-gCNV's default count-based filter excluded 17 genes. For contig ploidy priors, prior probabilities were set to 0.96 for ploidy state 2 and 0.01 for ploidy states 0, 1, 3, and 4. GermlineCNVCaller was executed in cohort mode, with the maximum copy number

state set to CN = 4. We note that GATK-gCNV was applied in this analysis outside its original intended scope, as the method was developed for detecting CNVs in uniquely mappable regions and by default excludes low-mappability exons commonly found in paralogous genes.

Figures and Tables

Gene	no. of samples	ExomeDepth (custom)	Edgecopy
SMN1/2 (1-6)	1,121	0.757	0.994
SMN1/2 (7-8)	1,121	0.682	0.988
C4A/C4B	45	0.992	1.000
FCGR3A/3B	123	0.826	0.869
Beta-defensin genes	106	0.736	0.994
RHCE/RHD	36	0.667	1.000
APOBEC3A/3B	174	0.732	0.977

Supplementary Table 1. Accuracy of exome copy number estimates for Edgecopy and ExomeDepth using experimentally derived copy number measurements for 6 paralogous genes. The values represent the proportion of samples for which the exome-based copy number estimate exactly matched the experimentally determined copy number. For *SMN1/2*, exons 1-6 and exons 7-8 were evaluated separately. Beta-defensin genes include six genes overlapping a single segmental duplication in the beta-defensin region (*DEFB107A*, *DEFB105A*, *DEFB106A*, *DEFB104A*, *DEFB103A*, and *DEFB4A*).

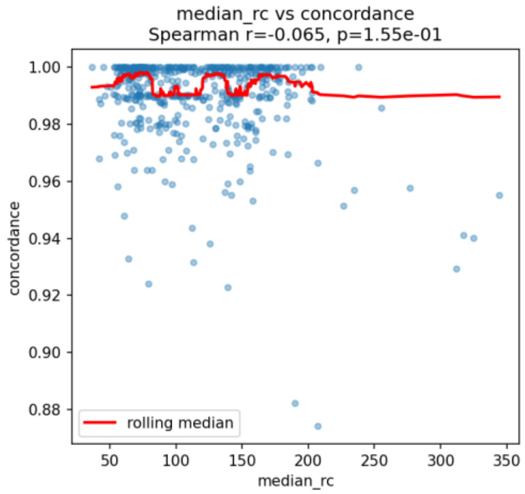

Supplementary Figure 1. Per-sample CN genotype concordance as a function of sequencing depth for 483 European ancestry samples across 108 paralogous genes. Each point represents a sample, with median read depth on the x-axis and concordance on the y-axis. The x-axis is the median per-exon read depth for each sample (normalized for exon length) calculated using non-duplicated exons. Spearman correlation analysis shows no significant correlation (p=0.155) between concordance and median read depth.
